# Stable and Reusable Lace-like Black Silicon Nanostructures Coated with Nanometer-Thick Gold Films for SERS-Based Sensing

**DOI:** 10.1021/acsanm.3c00281

**Published:** 2023-03-09

**Authors:** Lena Golubewa, Aliona Klimovich, Igor Timoshchenko, Yaraslau Padrez, Marina Fetisova, Hamza Rehman, Petri Karvinen, Algirdas Selskis, Sonata Adomavičiu̅tė-Grabusovė, Ieva Matulaitienė, Arunas Ramanavicius, Renata Karpicz, Tatsiana Kulahava, Yuri Svirko, Polina Kuzhir

**Affiliations:** † Department of Molecular Compound Physics, State Research Institute Center for Physical Sciences and Technology, Sauletekio Av. 3, Vilnius LT-10257, Lithuania; ‡ Department of Physics and Mathematics, Center for Photonics Sciences, University of Eastern Finland, Yliopistokatu 7, Joensuu FI-80101, Finland; § Department of Organic Chemistry, State Research Institute Center for Physical Sciences and Technology, Sauletekio Av. 3, Vilnius LT-10257, Lithuania; ∥ Department of Characterization of Materials Structure, State Research Institute Center for Physical Sciences and Technology, Sauletekio Av. 3, Vilnius LT-10257, Lithuania; ⊥ Institute of Chemical Physics, Vilnius University, Sauletekio Av. 9, Vilnius LT-10222, Lithuania; # Department of Physical Chemistry, Vilnius University, Naugarduko 24, Vilnius LT-03225, Lithuania

**Keywords:** black silicon, nanoroughness, surface-enhanced Raman spectroscopy, reusability, long-term stability, surface plasmon, doxorubicin

## Abstract

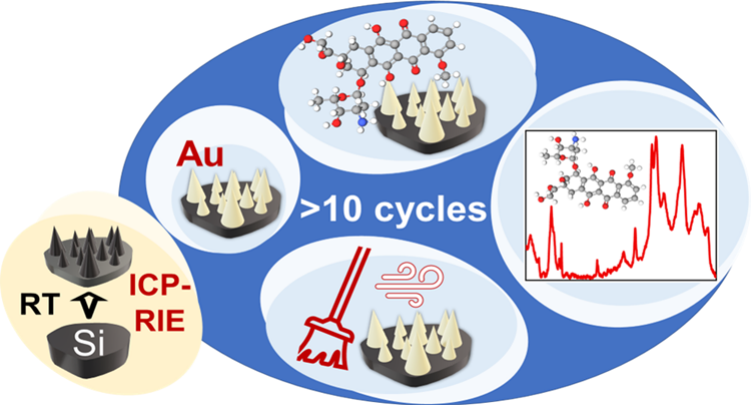

We propose a simple, fast, and low-cost method for producing Au-coated black Si-based SERS-active substrates with a proven enhancement factor of 10^6^. Room temperature reactive ion etching of silicon wafer followed by nanometer-thin gold sputtering allows the formation of a highly developed lace-type Si surface covered with homogeneously distributed gold islands. The mosaic structure of deposited gold allows the use of Au-uncovered Si domains for Raman peak intensity normalization. The fabricated SERS substrates have prominent uniformity (with less than 6% SERS signal variations over large areas, 100 × 100 μm^2^). It has been found that the storage of SERS-active substrates in an ambient environment reduces the SERS signal by less than 3% in 1 month and not more than 40% in 20 months. We showed that Au-coated black Si-based SERS-active substrates can be reused after oxygen plasma cleaning and developed relevant protocols for removing covalently bonded and electrostatically attached molecules. Experiments revealed that the Raman signal of 4-MBA molecules covalently bonded to the Au coating measured after the 10th cycle was just 4 times lower than that observed for the virgin substrate. A case study of the reusability of the black Si-based substrate was conducted for the subsequent detection of 10^–5^ M doxorubicin, a widely used anticancer drug, after the reuse cycle. The obtained SERS spectra of doxorubicin were highly reproducible. We demonstrated that the fabricated substrate permits not only qualitative but also quantitative monitoring of analytes and is suitable for the determination of concentrations of doxorubicin in the range of 10^–9^–10^–4^ M. Reusable, stable, reliable, durable, low-cost Au-coated black Si-based SERS-active substrates are promising tools for routine laboratory research in different areas of science and healthcare.

## 1 Introduction

Surface-enhanced Raman spectroscopy (SERS) technique relies on the enhancement of the electric field occurring when the frequency of an excitation beam coincides with the eigenfrequency of an ensemble of conduction electrons in a metallic nanostructured substrate.^1,2^ Experimentally obtained enhancement factors that vary from 10^4^ to 10^11^^3−5^ allow one to employ SERS to detect molecules with an extremely low Raman scattering cross-section of 10^–31^ cm^2^/sr^6^ and to reduce the detectable concentration of an analyte down to 10^–11^–10^–6^ M^7−9^ and even to attomolar values.^10^

SERS is widely recognized as one of the most efficient analytical techniques in organic chemistry and biochemistry. However, the implementation of SERS in practice still suffers from several limitations. Among the crucial ones are (i) the intrinsic inhomogeneity of SERS substrates, which results in a variation of the Raman signal over the substrate surface at a low concentration of the analyte, (ii) rather limited opportunities for quantitative analysis, (iii) poor long-term stability and thereby short exploitation time of SERS substrates, and (iv) their disposability.

Among systems commonly used for SERS are nanoparticles of copper/silver/gold stabilized in colloidal solutions. Even though colloidal solutions of noble metals exhibit very good enhancement of the Raman signal, their performance in SERS may be hampered by temperature variations and contamination with inorganic salts.^11^ The latter may lead to agglomeration of the metal nanoparticles, making the substrate no longer suitable for SERS.

Sacrificing the high enhancement, but greatly benefiting in repeatability, surfaces with immobilized noble metal nanoparticles,^12−14^ or metal-capped structured arrays^15^ and nanowires,^16^ Au-coated flexible polymer “fingers”,^17^ are increasingly used in laboratory practice. However, their fabrication techniques, such as electron-beam lithography,^18−21^ focused ion-beam lithography,^22^ and metal-assisted chemical etching,^23^ are often multistep, complex, and not cost-efficient, leaving room for the development of new approaches for cheap and scalable SERS substrate production.

In the search for SERS substrates, silicon has attracted special attention because silicon platforms of modern electronics open avenues for the development of the lab-on-chip approach. There are several methods of using silicon materials as precursors for SERS-active substrates. In ref (24), e-beam lithography is used to modify self-assembled monolayers of 3-(4-nitrophenoxy)-propyltrimethoxysilane on Si/SiO_2_ surfaces to push the assembly of citrate-passivated gold nanoparticles in distinct locations, creating patterned immobilization of Au NPs. Peng et al. applied metal-assisted chemical etching for the formation of micro-/nano-nested structures in Si with further Au deposition^25^ for the fabrication of a SERS microfluidic platform with a satisfactory EF of around 10^6^ and a prominent long-term stability of around 120 days. The fabrication approach of Si nanopillars capped with noble metals by the silicon plasma-etching method, omitting the lithographic step,^5^ resulted in the low-cost production of nanopillar arrays, providing significant enhancement (10^11^) and sensitivity.

However, there is always a trade-off among sensitivity, reproducibility, cost, stability (mechanical, operational, and durable), and reusability. As the laboratory routine usually involves multiple repeated experiments, the use of disposable SERS substrates leads to a corresponding multiplication of the resources needed. Cost and resource reduction can be achieved by developing large-scale and long-living substrates along with relevant methods for their reuse.

To address these challenges, we fabricated three-dimensional (3D) lace-type structures with a highly developed surface area over a 2 in. Si wafer using inductively coupled plasma reactive ion etching (ICP-RIE) technique at room temperature. These structures show a remarkably low reflectivity in the visual and near-IR spectral range^26^ belonging to a class of artificial materials referred to as black silicon (bSi). The bSi obtained at room temperature appears to be more ’porous’ and microrough^27^ than that fabricated at cryogenic temperatures, where precise control of the bSi surface shape, micropillar sidewall orientation, and roughness can be achieved via competing etching and passivation processes.^28,29^ The porosity of the bSi fabricated at room temperature allows one to increase the effective surface area and to achieve a higher—in comparison with regular micropillars—plasmonic “hotspot” surface density with the same amount of sputtered gold. In comparison with recently proposed gold–bSi structures,^30,31^ which contain 100–600 nm thick gold layers, we significantly reduce the amount of gold needed by creating a mosaic distribution of plasmonic hemispheres on the rich Si relief having a quasiperiodic lace-like structure. Moreover, the enhanced nanoroughness of the bSi surface enables plasmonic core–shell Si-Au nanostructures,^32^ rendering the buffer layer (e.g., Ti thin film), conventionally used to improve the adhesion of plasmonic nanoparticles, unnecessary.^29^

Analysis of the reliability, durability, stability, and reusability of Au-coated black Si-based SERS-active (bSi/Au) substrates reveals the beneficial “lace-type” bSi and mosaic gold geometries. Moreover, these “lace and mosaic” bSi/Au structures are much easier to manufacture, not requiring cryogenic conditions and being low cost due to the significant reduction in gold consumption. In addition, the stable Raman signal from the silicon underlying a very thin Au layer could be applied for precise calibration of peak intensities in the measured SERS spectra of analyte molecules, supporting capabilities of using such substrates not only for qualitative but also for quantitative analyte monitoring.

We suppose that the proposed simple methods of bSi/Au substrate fabrication and reuse, along with their applicability for monitoring trace amounts of molecules and their concentration determination, form a bridge for the translation of the SERS method from individual research laboratories for mass routine use in analytical research in many areas of biochemical and medical diagnostics, forensics, etc.

## 2 Methods and Materials

### 2.1 Materials and Reagents

A silicon ⟨100⟩ oriented wafer (p-type) with 1–20 Ω·cm (Si-Mat, Germany) was used for SERS substrate fabrication.

Analytical grade 4-mercaptobenzoic acid (4-MBA, Sigma Aldrich, Germany), ethanol (EtOH, >99.9%, Sigma Aldrich, Germany), and doxorubicin hydrochloride (DOX, Sigma Aldrich, Germany) were used throughout the experiments.

### 2.2 bSi/Au Fabrication

A 2-inch silicon wafer was etched using one-step mask-free inductively coupled plasma reactive ion-etching technique. The plasma etching was carried out in a PlasmaLab80 system (Oxford Instruments plc, UK). A two-gas mixture of SF_6_ and O_2_ with flow rates of 10 and 9 sccm, respectively, was used in the etching process. Dry anisotropic silicon etching was conducted for 10 min at 30 mTorr chamber pressure and an RF power of 15 W as well as 200 W ICP power. The cooling system temperature was set to 20 °C. Before the etching experiments, the silicon wafer was cleaned by oxygen plasma for 2 min.

**Figure 1:**
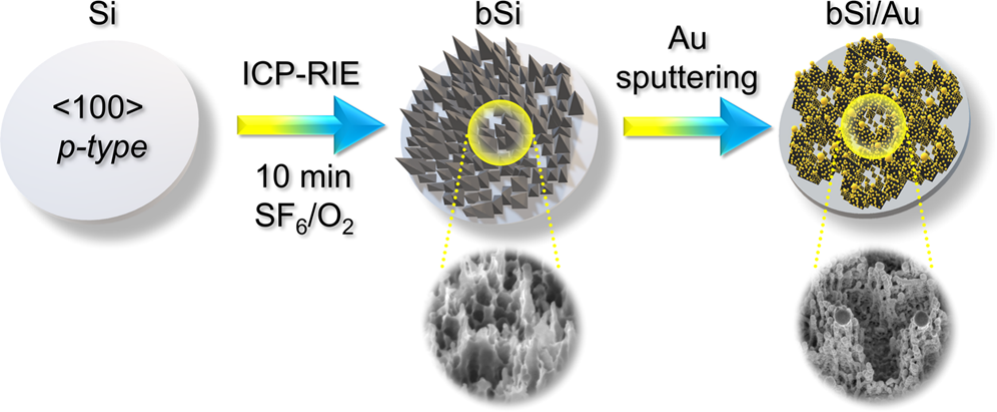
Steps of bSi/Au SERS substrate synthesis.

Gold deposition was carried out by magnetron sputtering for 80 s on top of the bSi substrates covered with a native oxide layer using the gold target (TARGET GOLD, 99.99%, 57 mm, 91017-AU, CAS 7440-57-5) purchased from Electron Microscopy Sciences (Hatfield, PA 19440). BSi/Au substrates sputtered simultaneously under the same conditions were stored in individual Petri dishes under normal ambient conditions for a period of up to 20 months, and each sample was analyzed immediately after the synthesis and after 1, 2, 3, 4, and 20 months of storage. The bSi/Au synthesis is schematically shown in Figure 1.

To evaluate the SERS performance of the fabricated bSi/Au substrates, we compare them with those obtained by the alternative cryogenic ICP-RIE technique.^28,32^ The surface morphology of the latter substrates is characterized by well-defined cone-like shapes. Correspondingly, the substrates covered with gold after magnetron sputtering for 80 and 120 s will be hereafter referred to as bSi/Au(conical) and bSi/Au*(conical).

### 2.3 Scanning Electron Microscopy (SEM) and Image Processing

SEM images of bSi and bSi sputtered with gold were obtained using a Helios NanoLab 650 (FEI) microscope. Analysis of bSi and bSi/Au was performed at a nominal beam voltage of 3 kV.

The bSi surface analysis was carried out using open-source software ImageJ.^33^ SEM images with a total area of 5.5 μm^2^ were analyzed. Six hundred and thirty-nine gold structures were identified from the images and ranged using Beeswarm plot statistics in OriginPro21 (OriginLab Corporation, USA). Quantitative evaluation of the effective bSi surface area was performed in Wolfram Mathematica 12 (Wolfram Research, Inc.). Side-view SEM micrographs were used to estimate the roughness of the bSi.

### 2.4 Raman and SERS Spectroscopy of 4-MBA

For SERS measurements, bSi/Au, bSi/Au(conical), and bSi/Au*(conical) substrates were incubated in 4-MBA solution of 1.0 mM concentration (in ethanol) for 4 h to allow the formation of a self-assembled monolayer (SAM) of 4-MBA molecules. Thereafter, each substrate was washed in a flow of ethanol to remove excess 4-MBA molecules and then rinsed in ultrapure water, dried in a laminar N_2_ flow, and used for measurements.

The Raman spectra were recorded using RamanFlex 400 (PerkinElmer, Inc.), equipped with a near-infrared 785 nm laser delivering 100 mW at the sample (maximum) and a high-sensitivity open-electrode CCD detector (air-cooled, operated at −50 °C). All Raman and SERS spectra were collected from at least three points of the sample surface and then averaged. The laser power on the sample was adjusted to 16.8 mW, and the illumination time was 300 s in all measurements except when analyzing the effect of the employed laser power and exposure time on the spectra of 4-MBA SAM on the bSi/Au substrate.

To evaluate the effect of the laser power and exposure time on the spectra of 4-MBA SAM on the bSi/Au substrate, the laser power was varied from 3.8 to 90.9 mW with a step of 4.6 mW and the accumulation time was linearly changed from 1 to 60 s.

The uniformity of the hotspot distribution was estimated with a laser-scanning MonoVista CRS+ Raman microscope system (S&I GmbH, Germany) equipped with a liquid-nitrogen-cooled CCD detector. Raman spectra were collected using a 785 nm wavelength emitting laser as an excitation source. An Olympus 50 × /0.8 NA objective was used for the excitation and collection of Raman spectra. The spectrometer was calibrated using a fundamental vibrational band at 520.7 cm^–1^ of a silicon wafer. The power delivered to the sample was adjusted to 48.5 μW. An area of 100 × 100 μm on bSi/Au samples with 4-MBA SAM was scanned with 1 μm step, and the accumulation time was 1 s.

### 2.5 EF Calculation

The enhancement factor (EF) was estimated by the following equation^34^
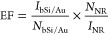
1where *I*_bSi/Au_ and *I*_NR_ are intensities of the SERS and normal Raman bands, and *N*_bSi/Au_ and *N*_NR_ are the numbers of molecules in 4-MBA SAM on the bSi/Au substrate and 4-MBA on the flat silicon surface, respectively.

### 2.6 Oxygen Plasma Cleaning of bSi/Au Substrates

Removal of the molecules attached to the bSi/Au substrate was carried out using oxygen plasma treatment. An oxygen plasma etching device built in Super Cool Sputter Coater Leica EM SCD050 (Leica Microsystems GmbH, Germany) was used for sample cleaning. bSi/Au substrates with a SAM of 4-MBA were placed in the load-lock chamber of a vacuum system. The parameters were set as follows: pressure 0.1 mbar and current 48 mA. The duration of oxygen plasma treatment was determined experimentally and corresponded to a >99% decrease in the intensities of the 1076 and 1588 cm^–1^ bands. Afterward, the substrates were washed with deionized water and ethanol, dried in N_2_ laminar flow, transferred to individual Petri dishes, and stored under ambient conditions before the next cycle of bSi/Au substrate reuse.

## 3 Results and Discussion

### 3.1 bSi/Au Surface Characterization

**Figure 2:**
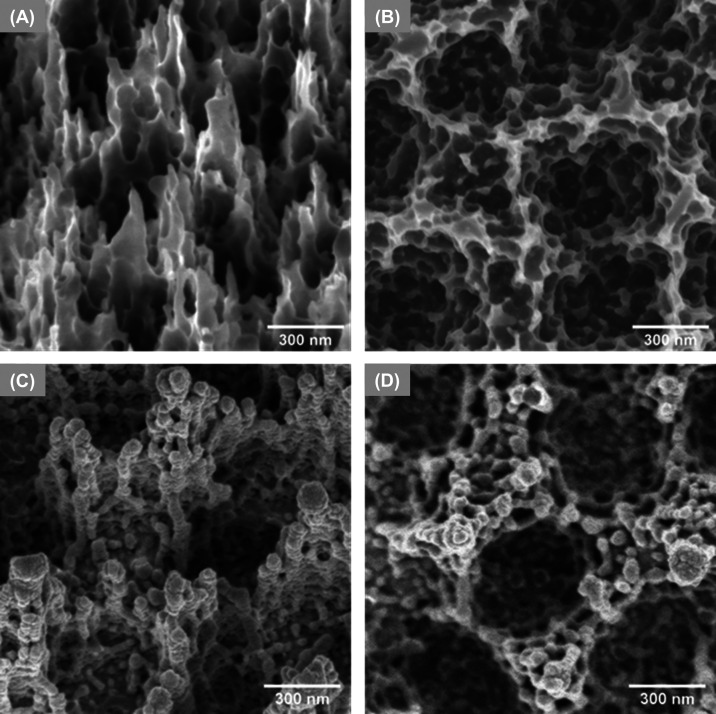
SEM micrographs of bSi (A, B) and bSi sputtered with gold (C, D): angle-side (A, C) and top–bottom (B, D) views.

SEM images of the obtained bSi/Au substrate are presented in Figure 2. The surface morphology is characterized by vertically standing, thin, lace-like, sharp-edged structures (Figure 2A), with an average height of around 1 μm and a base of 100–200 nm. These plates form wells with an average diameter of 500 nm (Figure 2B), the inner surface of which is also covered with rock-like structures with sharp peaks with high apex curvature, but their dimensions are much smaller and are around 10–100 nm. These various micro–nano structures are responsible for the very low light reflection in the visible and near-infrared (NIR) ranges and appear completely black to the naked eye.^26^ The synthesized bSi demonstrates high absorption in the range of 200–900 nm (see Figure S1, Supporting Information). Numerous micro–nano structures comprising bSi surfaces create light traps that drastically increase the absorbance. This light-trapping ability of bSi in the vis–NIR range also drastically contributes to the excitation of the collective electron oscillations in the deposited gold particles, the phenomenon responsible for the high enhancement of the Raman signal in SERS.^35^

The fabrication process allowed us to obtain large-scale bSi substrates with structures uniformly distributed over a 2 in. Si wafer (see Figure S2, Supporting Information).

The micro–nano-structured bSi was then sputtered with gold to obtain plasmonic structures densely distributed over the bSi. SEM micrographs of bSi/Au are presented in Figure 2C,D. Side walls of vertically oriented Si plates are covered with gold hemispheres of sizes (diameters) from 4 to 16 nm (10.8 nm on average), while the sharp apexes of these plates serve as nuclei for spherical NP formation of sizes from 16 to 60 nm (34.6 nm on average). Also, in some cases, spherical NPs grew to sizes from 60 to 195 nm (109.7 nm on average) (see Figure S3, Supporting Information). The essential differences between these bSi/Au structures and bSi/Au (conic) substrates^30−32^ are the significantly reduced Au deposition, which resulted in a very thin gold layer (tens versus hundreds of nanometers) and the presence of gold-free zones, which, as it will be shown below, can be used for independent signal normalization and quantitative determination of the concentration of the studied substances.

### 3.2 bSi/Au Enhancement Factor Calculation

To evaluate the effectiveness of the bSi/Au substrate for SERS, 4-mercaptobenzoic acid (4-MBA) was used as a test molecule. 4-MBA is widely utilized as a standard because its molecules form a self-assembled monolayer of well-known surface density and Raman spectrum.^36−38^ BSi/Au substrates were incubated with 4-MBA as described in the Methods and Materials Section, which resulted in the formation of covalent bonding of 4-MBA through the thiol group with gold, with the creation of a S–Au bond.

**Figure 3:**
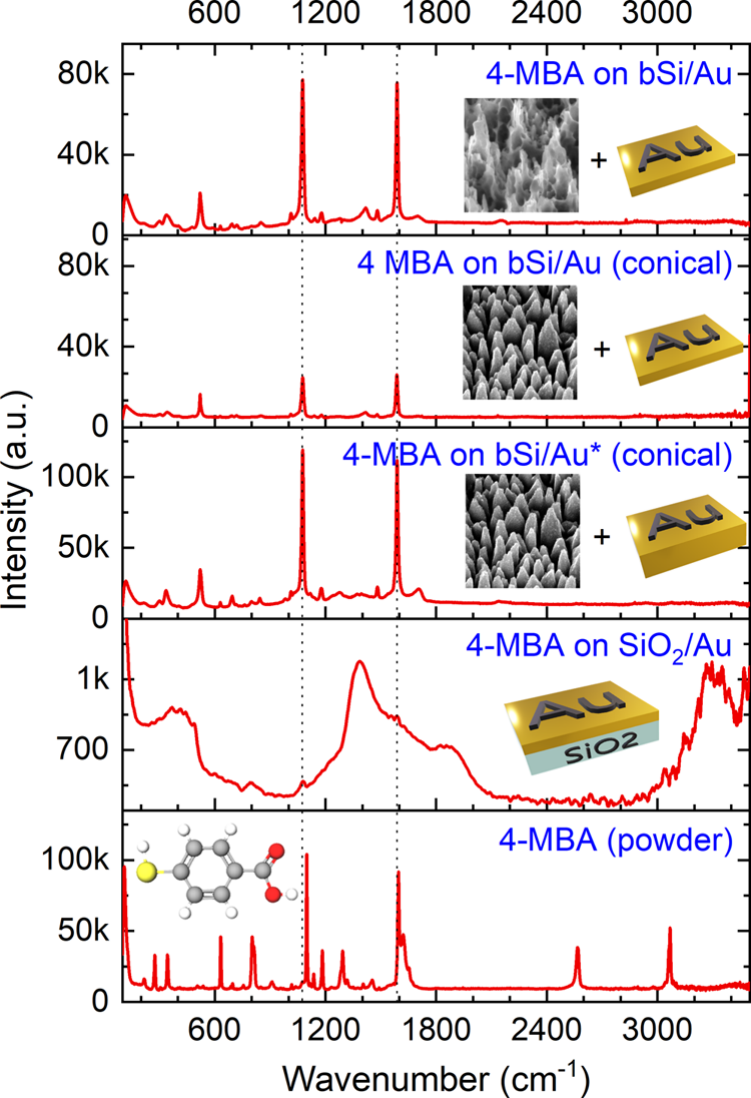
Raman spectrum of 4-MBA powder (bottom) and SERS spectra of 4-MBA on SiO_2_/Au, bSi/Au* (conical),^32^ bSi/Au (conical), and bSi/Au substrates (from bottom to top, respectively). *30% thicker gold layer sputtered on the surface of bSi than in other cases (bSi/Au (conical) and bSi/Au).

SERS spectra of 4-MBA on the bSi/Au and SiO_2_/Au substrates and the Raman spectrum of bulk 4-MBA are presented in Figure 3. bSi/Au substrates display a significant Raman intensity increase as compared with the SiO_2_ substrate with nanostructured gold deposited on it. The assignment of characteristic bands of 4-MBA is presented in Table S1 (Supporting Information). As shown in Figure 3, several bands of 4-MBA molecules are significantly enhanced according to the surface selection rules:^39^ 1076 cm^–1^ ν_12_ (a_1_) aromatic ring breathing mode and 1587 cm^–1^ totally symmetric aromatic ring vibration (ν_8a_). The intensive band in the Raman spectrum of bulk 4-MBA at 2570 cm^–1^, which arises from the S–H stretching in 4-MBA, disappears in the SERS spectrum of 4-MBA on the bSi/Au substrate, while a new band around 260 cm^–1^ appears, indicating the breakage of the S–H bond in the 4-MBA molecule and the formation of a S–Au covalent bond^36,39^ and the 4-MBA self-assembled monolayer. A band at 521 cm^–1^ associated with Si is also present in the recorded spectra, as a thin mosaic cover of irregular nanoscale gold islands does not eliminate this band.

Bands associated with the carboxylic moiety of the 4-MBA molecule are present in the spectrum of the 4-MBA SAM on the bSi/Au substrate: the bands at 849 and 1417 cm^–1^ correspond to bending β(COO^–^) and stretching ν(COO^–^) vibrations and indicate the presence of deprotonated carboxylic groups, and the band at 1702 cm^–1^ arising from ν(C=O) indicates the presence of protonated carboxylic groups.^39^ The normalized intensity of the ν(COO^–^) band to ν_8a_ is often used to determine the pH value as this ratio increases with the growth of the deprotonation level of 4-MBA molecules.^40^ However, in dried samples of bSi/Au with 4-MBA, the presence of β(COO^–^) and ν(COO^–^) vibrations in the SAM more likely indicates the direct interaction of COO^–^ groups with the metal surface, which forces the flat orientation of the aromatic ring of 4-MBA molecules.^38^ This assumption is supported by the appearance of γ(CCC) out-of-plane ring vibrations at 718 cm^–1^^39^ and a weak aromatic ν(CH) stretching band at 3074 cm^–1^ in SERS (see Table S1, Supporting Information), which weaken for flat-oriented aromatic rings due to surface selection rules.

It is worth noting that there are no characteristic signs of the decarboxylation process, which is caused by the interaction of 4-MBA molecules with plasmonic active surfaces and may be a result of the excessive surface irradiation and subsequent heating along with molecular degradation.^41^ Decarboxylation is indicated by the appearance of two new peaks at 996 and 1019 cm^–1^ associated with benzene monosubstituted thiophenol.^38^ In the case of the 4-MBA SAM on the bSi/Au substrate, no decarboxylation occurs, as only the 1012 cm^–1^ band assigned to ring deformations is present in the SERS spectra.

We compared the Raman signal enhancement for bSi/Au and bSi/Au (conical) substrates. The morphology of the latter substrate, which was described in ref (32). is dominant, with mostly smooth conical structures of 500 nm height and 200 nm base. From the fabrication point of view, the principal difference between these bSi/Au and bSi/Au (conical) substrates is that a transition from the cryogenic ICP-RIE to room temperature (RT) ICP-RIE occurred, which simplified and reduced the cost of fabrication of bSi and, at the same time, resulted in a more developed surface area (so-called lace-like bSi structures appeared). As it follows from Figure 3, cone-like silicon structures of bSi/Au* (conical) provide significant Raman signal enhancement. However, a reduction in the gold layer thickness by 30% leads to a decrease in the intensity by 4.6 times. The deposition of a gold layer of the same reduced thickness on the lace-type bSi with sharp lace-like structures gives a 3 times more intensive signal than bSi/Au (conical) with the same gold layer thickness (see Figure 3). This significant increase is due to the more developed surface area, which enables a larger number of hotspots. It is also important to note that either overlapping of nearby gold nanoparticles or the formation of vertically oriented dumbbell-like structures of the characteristic sizes obtained under the employed conditions of gold deposition (10.8 nm for side walls of Si plates and 34.6–109.7 nm for apex caps), as well as their vertical ordering on plates of bSi of the considered profile, also significantly contribute to the incidence of the plasmon resonance frequency of the substrate under consideration in the near-IR region, as previously demonstrated in ref (32). As a result, a successful combination of the profile of the supporting substrate and the appropriate size and orientation of the systems of gold nanoparticles allows one to consider the produced substrate as an effective material for sensitive SERS-based analyte detection using NIR excitation, which is extremely important for biomedical and biochemical tasks due to the safety of NIR irradiation as compared with UV light.

Further, the enhancement factor of the bSi/Au substrate was calculated using eq 1 (Methods and Materials) for the band at 1076 cm^–1^, with reference to the Raman spectra of 4-MBA on the Si wafer. The effective surface area of the synthesized bSi sputtered with gold was calculated from SEM micrographs and is at least 20 times larger than that of bSi/Au (conical) substrates (based on estimations presented in Section 5, Supporting Information). The EF of the bSi/Au substrate was estimated to be around 1.1 × 10^6^; calculations are given in the same section of the Supporting Information.

Enhancement of the Raman signal in SERS occurs through two possible mechanisms: electromagnetic (EM) and chemical (CM). These two mechanisms could be realized together or separately. The EM mechanism is based on the phenomenon of plasmon resonance and occurs when the frequency of the exciting radiation coincides with the frequency of collective oscillations of delocalized conducting electrons (or surface plasmons, SPs), which creates an electromagnetic field on the metal nanostructure/dielectric interface. The EM mechanism has the largest impact on signal enhancement (up to 10^11^),^42^ while CM manifests only a 10^0^–10^3^ times intensity increase^43^ due to the charge-transfer (CT) process.^44^ The calculated EF for BSi/Au has a magnitude of 10^6^, indicating that the major impact on the SERS signal comes from the EM mechanism. However, CT could not be excluded as its possibility was demonstrated previously in ref (32) by DFT simulations for similar structures.

As it follows from a comparison of the obtained EF with the EF calculated for the bSi/Au* (conical) substrate,^32^ there is a 2 order difference in EFs (10^6^ for bSi/Au vs 10^8^ for the foregoing bSi/Au* (conical)), while the intensity values of the 1076 cm^–1^ band of 4-MBA molecules for both substrates are of the same order, specifically *I*_SERS_ (on bSi/Au lace-like) ≈2/3 *I*_SERS_(on bSi/Au* (conical)). This apparent inconsistency can be explained as follows. EF is calculated per molecule and strongly depends on the Au layer thickness, while measured intensities are determined by the joint contribution of irradiated molecules, the number of which is strongly dependent on the surface area available for molecular attachment, the hotspot density, and the E-field enhancement. Thus, a decrease in Au layer thickness leads to a decrease in the electromagnetic field enhancement, and an increase in the adsorbed molecules leads to a decrease in the *I*_SERS_/*N*_SERS_ multiplier in Formula 1, and all of these result in a decrease of 2 orders in EF (as it is calculated per molecule) in comparison with the EF of bSi/Au* (conical). On the other hand, in the case of the detected spectra when the signal is provided by the additive impact of molecules trapped in the laser beam, due to the increased hotspot density and still satisfactory EM enhancement, the measurements of both bSi/Au and bSi/Au* (conical) provide comparable intensity values. This outcome is an obvious advantage of the bSi/Au substrate, described in the present study, as it is much cheaper and easier to fabricate than its foregoer bSi/Au* (conical).

The EF, demonstrated by micro-nano-structured bSi/Au substrates, is comparable to that reported for various SERS-active nanostructures, such as gold,^45^ silver,^46^ or gold–silver core–shell nanorods,^47^ nanostars,^48^ nanoflowers,^49^ etc. Although it is lower than that for Ag-aligned^50^ or semiconducting 15%-Mo-Ta_2_O_5_^51^ nanorods (10^6^ vs 10^8^ and 10^7^, respectively), bSi/Au has the significant advantage of being fabricated by the simple, fast, and low-cost RT-ICP-RIE technique. Moreover, the spontaneous formation of regular silicon lace-like structures with nanoroughness, which serve as precursors for the hotspots, excludes the most complex and time-consuming stage of large-scale fabrication of uniform and homogeneous substrates—the immobilization of nanostructures on a carrier surface (for example, quartz). The nanorough lace-like shape of the bSi/Au substrate also makes it very similar in geometry to needle-shaped regular nanostructured arrays, which were successfully applied for the SERS-based detection of SARS-CoV-2 virus^52^ with extremely high affinity, where human angiotensin-converting-enzyme 2-functionalized gold needles served as effective virus traps. This similarity in geometry may expand the application of bSi/Au to the most urgent and vital areas of bioresearch and allow further consideration of the bSi/Au SERS substrate as a solution when emergencies, such as the coronavirus pandemic, require fast, large-scale, low-cost, and effective actions.

### 3.3 bSi/Au Responsivity, Photothermal Effect (PTE), and Heating Limitations

**Figure 4:**
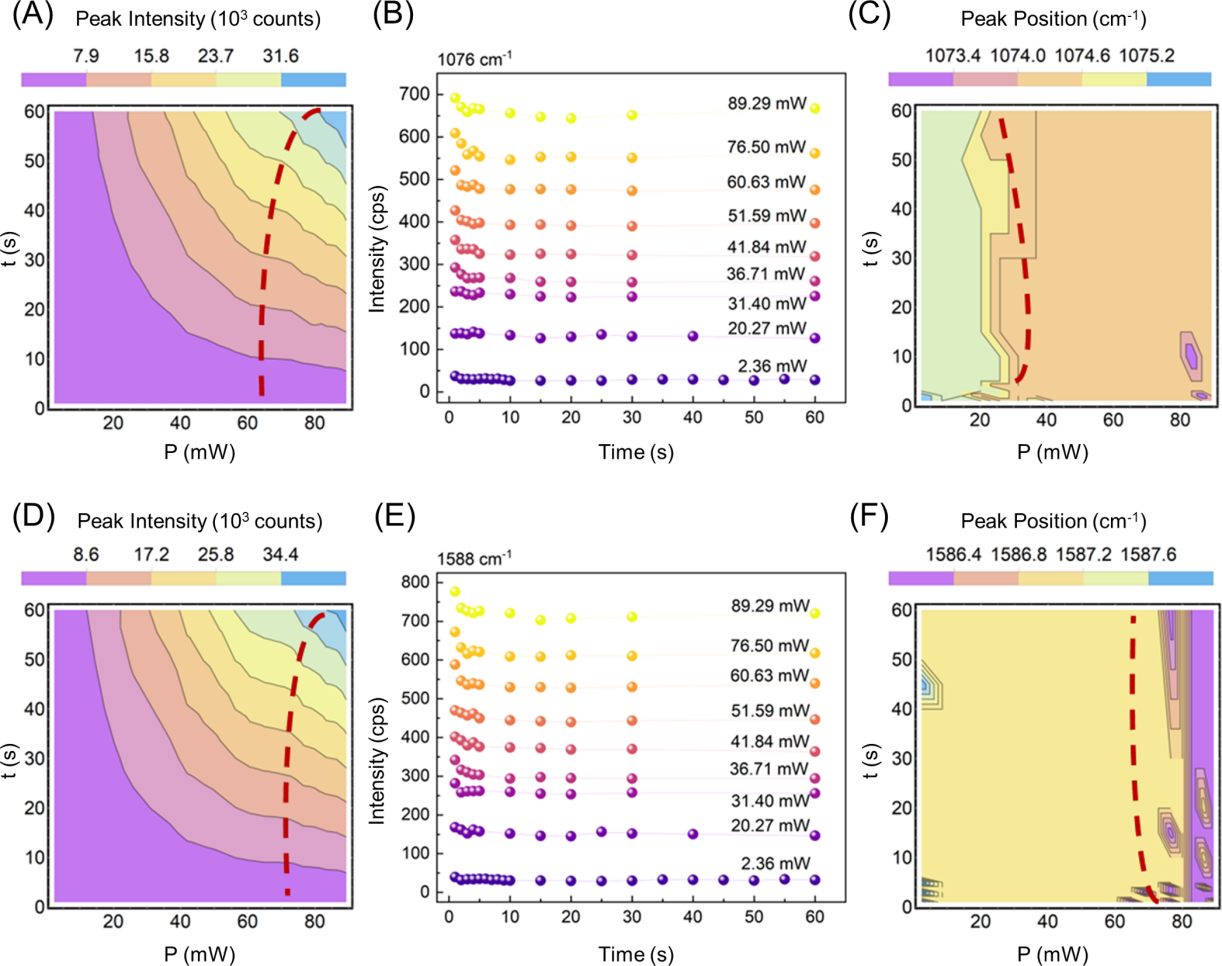
Evaluation of the photothermal effect on characteristic bands in SERS spectra of 4-MBA SAM on the bSi/Au substrate. SERS peak intensities of 1076 (A) and 1588 cm^–1^ (D); dynamic curves of intensities (in cps) obtained for 1076 cm^–1^ (B) and 1588 cm^–1^ (E) bands; the dependencies of the peak positions of 1076 cm^–1^ (C) and 1588 cm^–1^ (F) bands on the laser power and time of exposure of the probed 4-MBA molecules to excitation irradiation. The excitation wavelength is 785 nm. Ranges on the false color maps that correspond to photothermally affected and unaffected spectral characteristics are separated by red dashed lines.

To estimate the possible substrate heating that might affect the efficiency of the proposed SERS substrates, the SERS spectra of a 4-MBA monolayer on bSi/Au were obtained, varying the laser power from 3.8 to 90.9 mW and sample exposure time from 1 to 60 s. Variation of the intensities at 1076 and 1588 cm^–1^ and their shifts are presented in Figure 4. The bSi/Au substrate provides a detectable signal already at 1 s of exposure with 3.6 mW laser power, and it reaches values suitable for analysis after 7 s of irradiation at 13 mW laser power. An intensity vs time SERS signal profiling revealed a slight decrease in SERS measured at both 1076 and 1588 cm^–1^ when the total energy imparted to the sample reaches 70 mW × 20 s = 1.4 J (see Figure 4A,D).

Local heating of gold plasmonic nanostructures may negatively affect the EF, lead to a decrease in the SERS signal,^53,54^ and cause photothermal damage to the analyte, resulting in its degradation.^55^Figure 4B and E show the dynamics of the 1076 and 1588 cm^–1^ band intensities (in cps) in the SERS spectra of the 4-MBA SAM at different laser powers and exposure times of the probed molecules to irradiation at λ_ex_ = 785 nm. As can be noticed from the dynamic curves, there is no significant decrease in the SERS intensity under a wide range of experimental conditions, and only at laser powers exceeding 31.4 mW, a decrease of less than 10% in the intensity occurs. The results indicate that in contrast to, e.g., Mo-doped Ta_2_O_5_ semiconductor SERS substrates,^51^ for which photoinduced degradation is non-negligible and could not be ignored as 5 mW and 60 s irradiation of 10^–6^ M methyl violet with a 532 nm laser causes approx. 30% intensity loss, bSi/Au SERS substrates could be used in wider time and laser power scales. The peak shift dependence on the laser intensity detected in Figure 4C,F may also correspond to the possible sample damage due to the high laser intensities, which was not detected using SERS intensities of the most intensive bands (1076 and 1588 cm^–1^).

**Figure 5:**
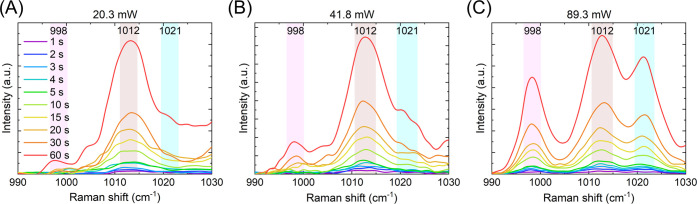
SERS spectra of a 4-MBA monolayer on bSi/Au in the range of 990–1030 cm^–1^. The exposure time varies from 1 to 60 s. The excitation powers are (A) 20.3, (B) 41.8, and (C) 89.3 mW.

Analysis of the 990–1030 cm^–1^ range also reveals the PTE in 4-MBA SAM on the bSi/Au surface. Bands at 996 and 1021 cm^–1^, which are associated with monosubstituted benzene derivatives (out-of-plane and in-plane ring deformations, respectively^56^), appear at the excitation power of 41.8 mW and exposure time of 20 s (see Figure 5). As mentioned above, the appearance of monosubstituted benzene derivatives indicates that the decarboxylation of the 4-MBA molecule occurs. The loss of the carboxylic group is caused by the plasmon-derived “hot” electron transfer from the metal to the absorbed molecules because of the plasmon excitation and is promoted by substrate heating. The obtained results allow assuming the upper limit for the total energy delivered to the analyte, avoiding the PTE, to be close to 1.0 J.

### 3.4 bSi/Au Substrate Uniformity

**Figure 6:**
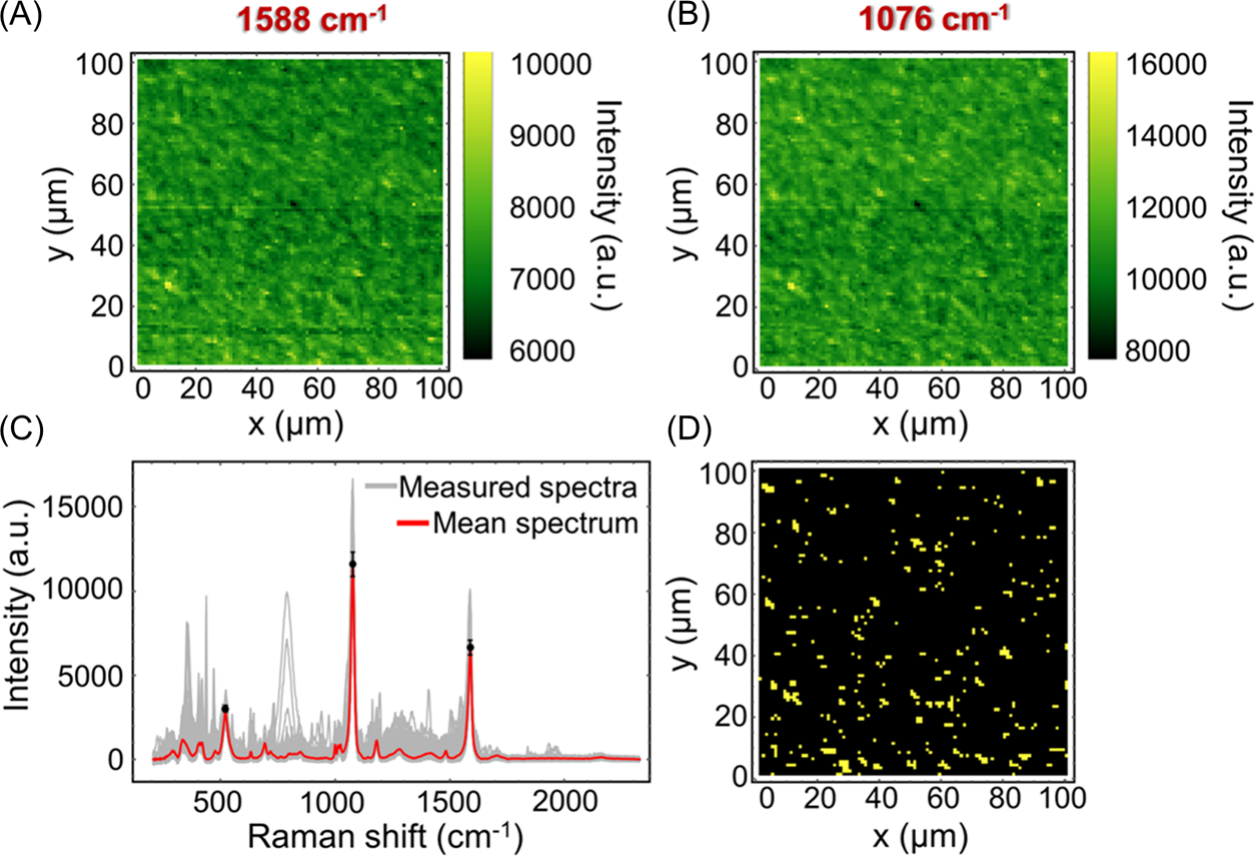
Two-dimensional (2D) distribution of the peak intensities at 1076 (A) and 1586 cm^–1^ (B) of SERS spectra of the 4-MBA monolayer deposited on top of the bSi/Au substrate. (C) 4-MBA SERS spectra from the whole scanned area of 100 × 100 μm^2^ (shown in gray color); an orange spectrum corresponds to the mean SERS spectrum of 4-MBA. (D) Spatial distribution of accidental impurities. Measurement parameters: λ_ex_ = 785 nm, exposure time is 1s, laser power is 0.05 mW, scanning step is 1 μm, 50× objective.

Evaluation of the bSi/Au SERS-substrate uniformity in terms of signal enhancement was performed by laser-scanning confocal Raman microscopy. False color maps of the distribution of intensities of 1076 and 1588 cm^–1^ bands are presented in Figure 6A,C, respectively. Relative standard deviations for both peak intensities were 0.06, and the medians almost completely coincide in magnitude with the mean values (see Table S2, Supporting Information). These parameters indicate very good uniformity of the substrate and a high density of hotspots (scanning step was 1 μm) across the analyzed area of 100 × 100 μm^2^. However, in addition to the spectra of the analyzed analyte (4-MBA), spectra of impurities were detected, which were present on the substrate because of preparatory manipulations and/or came from the environment during the measurement itself (the gray spectra in Figure 6C). Additional statistical analyses showed that the number of “impurity” spectra was 434 out of 10201 (4%) (Figure S5, Supporting Information), impurities are distributed randomly and evenly over the substrate (see Figure 6D), and they can be easily identified and excluded from further analysis, significantly increasing the accuracy of the research.

### 3.5 Long-Term Stability of the bSi/Au SERS Substrate and its Reusability

**Figure 7:**
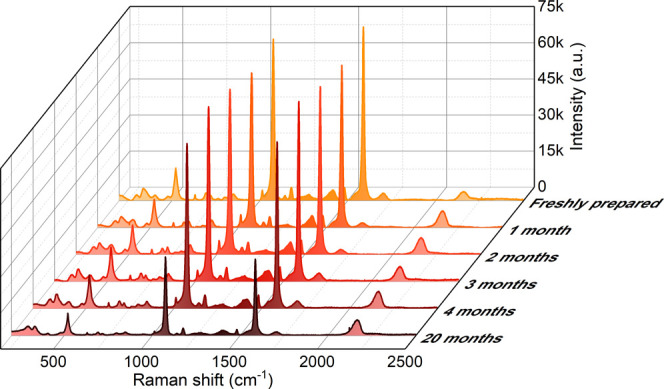
Storage stability of the bSi/Au substrate evaluated with 4-MBA as a probe analyte. Each SERS spectrum corresponds to a 4-MBA monolayer on the surface of freshly manufactured bSi/Au samples and bSi/Au samples stored for 1, 2, 3, 4, and 20 months.

Several simultaneously fabricated bSi/Au substrates were individually packed in plastic Petri dishes, wrapped with a parafilm to avoid accidental contamination, and stored under ambient conditions. These substrates were used to obtain 4-MBA SERS spectra after storage for 1–20 months in the same measurement setup. The results are summarized in Figure 7. The intensity of two characteristic bands at 1076 and 1588 cm^–1^ in the 4-MBA spectra decreased by less than 5% after 120 days and by 40% after 20 months of storage.

SERS spectra are collected from the molecules in the proximity of the bSi/Au surface. This is the main barrier to reusing the substrate, as physically or chemically adsorbed molecules are hardly eliminated. Remaining residues may decrease the effective area accessible for analyte molecules and yield signals that will interfere with the signal from the analyte; moreover, the cleaning procedure itself may damage the hotspots, decreasing the effectiveness of SERS spectra detection.

**Figure 8:**
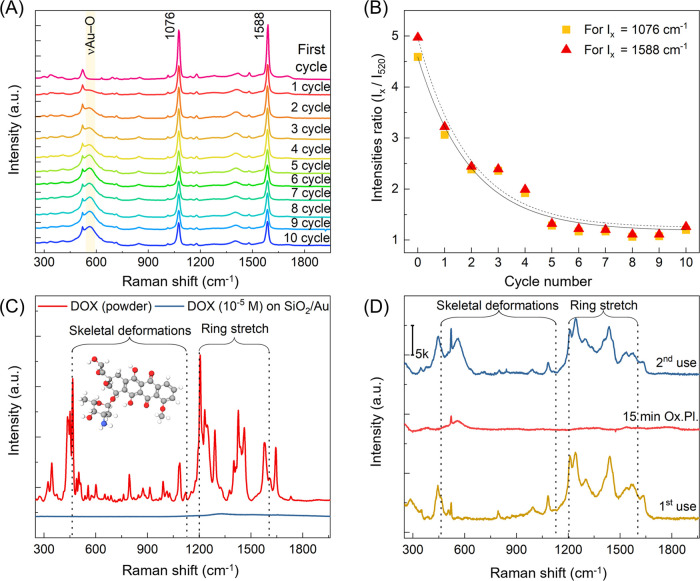
Versatility of the bSi/Au substrate reuse demonstrated for (A, B) covalently bonded molecules of 4-MBA and (C, D) noncovalently bonded molecules of DOX. (A) SERS spectra of 4-MBA on the bSi/Au substrate over 10 reusability cycles; (B) dependence of the 1076 and 1588 to 521 cm^–1^ intensity ratios on the number of reuse cycles; (C) Raman spectrum of DOX (powder) and SERS spectrum of 10^–5^ M of DOX on SiO_2_/Au; (D) SERS spectrum of 10^–5^ M of DOX on bSi/Au substrate before oxygen plasma cleaning (mustard line), spectra of bSi/Au substrate after 15 min of oxygen plasma treatment (red line), and SERS spectrum of 10^–5^ M DOX on the reused bSi/Au substrate (blue line).

To reuse the bSi/Au substrate, mild oxygen plasma cleaning at room temperature was chosen as a surface refreshing procedure due to its wide accessibility in laboratories. A decrease in the intensity of the most intense bands of 4-MBA at 1076 and 1588 cm^–1^ by more than 99% was chosen as a determining criterion for whether the bSi/Au surface was free of the analyte molecule. It was estimated that 35 min in oxygen plasma is enough for residue elimination from the bSi/Au substrate (see Figure S6, Supporting Information), and after rinsing the substrate in deionized water and drying, it can be reused. The recycling of the substrate leads to a 4-MBA signal decrease and an increase in the intensity of the band centered at 560 cm^–1^ and assigned to ν(Au-O) formation due to the oxygen plasma effect (see Figure 8A).^57^ To estimate the degree of EF decrease, the intensities of the 1076 and 1588 cm^–1^ bands were normalized with respect to the intensity of the 520 cm^–1^ band of silicon for each cycle of reuse. The intensity of the 520 cm^–1^ band remains constant over the whole area of the bSi/Au substrate (see Table S2, Supporting Information). As it does not change during the cleaning of the substrate with oxygen plasma, it is convenient to use this band as a reference to exclude possible laser intensity fluctuations in different experiments.

As it follows from Figure 8B, the intensity ratios decrease 5 times after the fifth cycle of reuse; however, the EF as compared with SiO_2_/Au remains acceptable for analyte detection and analysis. Moreover, the high uniformity of hotspot distribution allows the accumulation and further integration of the SERS signal from large-scale areas, which is of even higher importance than EF for detecting trace amounts of analytes.^10^

### 3.6 Evaluation of Doxorubicin Concentration with the bSi/Au SERS Substrate

The bSi/Au substrate has been proven to be suitable also for the detection of molecules that do not form covalent bonds with it. BSi/Au was applied to detect the spectrum of the anticancer drug doxorubicin (DOX).^58^ DOX causes many side effects, such as heart damage (cardiotoxicity), cardiomyopathy, and heart failure.^59,60^ Therefore, control of the DOX concentration in the blood plasma during chemotherapy is important to minimize the possible harm to the patient during treatment.

**Figure 9:**
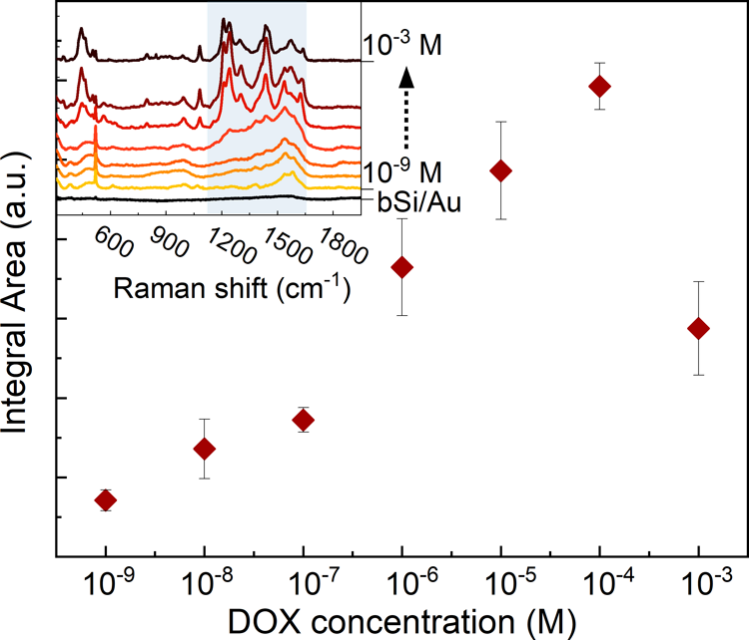
Calibration plot for the determination of DOX concentration by the bSi/Au substrate. Inset: SERS spectra of DOX at different concentrations (1.0 nM to 1.0 mM).

DOX has a characteristic Raman spectrum; however, it is challenging to detect low concentrations of DOX in water solutions (Raman spectra of DOX powder and 10^–5^ M DOX solution are shown in Figure 8C). This obstacle can be overcome by the SERS approach, allowing the detection of DOX at concentrations of 1.0 nM to 0.1 mM (see Figure 9). SERS spectra of a 10^–5^ M DOX solution are presented in Figure 8D (mustard line). Because of the low intensities of the R–H stretching range around 3000 cm^–1^, data are presented for the fingerprint region of 300–1800 cm^–1^. Characteristic vibrational frequencies of DOX are marked in the Raman and SERS spectra in Figure 8C,D; major band assignments are presented in Table S3 (Supporting Information).

The spectra of DOX were obtained from three different points with minor intensity fluctuations detected. The range 1150–1700 cm^–1^ corresponds to the ring stretch,^61^ and the areas under the spectra in this range were used to obtain the concentration calibration curve (see Figure 9). Violation of the monotonicity of the calibration curve at DOX concentrations >10^–3^ M is associated with two factors: (i) agglomeration of DOX molecules at high concentrations leading to changes in the spectrum; (ii) decay of the SERS signal with an increase in the distance from the plasmonic structures due to the formation of several layers of analyte molecules. Thus, the DOX concentration range detectable with bSi is between 10^–9^ and 10^–4^ M.

To regenerate the bSi/Au surface before the next measurement, it is sufficient to treat the surface with oxygen plasma for 15 min to completely remove all adsorbed DOX molecules. During continuous multiple SERS-based measurements of DOX by the bSi/Au substrate, the same intensities of spectrum bands are observed (see Figure 8D), although the ν(Au-O) band is detected and should be considered when reusing the bSi/Au substrate during continuous measurements.

## 4 Conclusions

Analytical application of SERS substrates implies long-term stability and reusability. The majority of the manufactured substrates are disposable. Therefore, they can be stored only for a short period of time because of the aggregation and precipitation of colloidal nanoparticles in solutions due to their extreme sensitivity to storage conditions,^62^ external environment, and/or impurities, due to the oxidation of metal nanoparticles, predominantly Ag and Au, immobilized on the supporting substrate,^63^ or because of the mechanical fragility of the structures supporting nanoparticles, such as silicon nanowires,^23^ and the irreversible collapse and agglomeration of plasmonic noble metal “caps” at the ends of flexible nanopillars^5,64^ caused by surface tension upon contact with the analyte.

In the present study, we propose a simple one-step production of bSi with an extremely high effective surface area by room-temperature ICP-RIE technique. The sharp vertically oriented lace-like silicon plates create favorable conditions for the growth of gold nanostructures, which are similar in shape to overlapping hemispheres of 4–16 nm sizes on the side walls of blades and spherical nanoparticles of around 34.6 nm on the apex of cones with several extralarge particles of average size around 109.7 nm. The created structures demonstrate high Raman signal enhancement with excitation in the NIR region (785 nm), with an EF of around 10^6^. A significant advantage of the produced bSi is the reduced thickness of the gold layer as compared with other gold-based structures used in SERS-based analyses.^30,31^ The high surface density of vertically aligned gold nanostructures ensures the shift of the absorption cross-section to the NIR range and the high magnitude of electromagnetic field enhancement, as demonstrated in several studies.^32,65^

The bSi/Au substrate is characterized by the high reproducibility of the results and low signal variation within 6% over a 100 × 100 μm^2^ area. Substrate storage in the air atmosphere and under normal ambient conditions results in less than 3% signal decrease over 4 months and around 40% decrease over 20 months of storage, which is, according to our knowledge, the longest term of SERS substrate storage, which offers good SERS signal enhancement. Along with this, the produced substrates can be reused. Substrate treatment with oxygen plasma, commonly available in most laboratories, allows one to get rid of the layer of molecules attached to the surface and retains the properties of the structures to enhance significantly the Raman signal from the analyte when the substrate is reused during continuous measurements. The number of reuse cycles exceeds 10. As proof of the concept, the SERS substrate was applied to detect the chemotherapeutic drug, DOX, and it was determined that DOX concentrations can be determined in the range of 1.0 nM to 0.1 mM. The developed bSi/Au surface recycling procedure preserved the sensitivity of the substrate toward DOX with similar sensitivity as no observable SERS signal decrease was registered. Moreover, such a thin gold layer (tens of nanometers) deposited on the highly developed surface of bSi does not eliminate the first-order Si Raman band in the SERS spectra of analytes, and this can be used for independent Raman signal normalization at the silicon line and thereby allows quantitative determination of the concentration of the studied substances.

Summarizing, the type of bSi proposed in this study is easily producible on a large scale as the nano-microstructure-based morphology can significantly reduce the cost because much lower amounts of noble metal (gold) are required for the formation of plasmonic structures. The bSi/Au substrate provides a significant signal enhancement upon excitation in the near-IR range with a reproducible, stable, and uniform signal. bSi/Au is a highly sensitive SERS substrate, which can be repeatedly used to detect trace amounts of analytes and can be effectively used to assess the concentration of analytes in aqueous media, for example, organics-based medicinal compounds. All of the above open the way for the mass application of bSi/Au SERS substrates in many fields of science, allowing their commercialization and wide use as a routine technique that does not require high costs, compliance with special (sterile, for example) conditions, and complex sample preparation.

## Supporting Information Available

Black silicon absorbance; large-scale uniformity and regularity of bSi structures; evaluation of gold nanoparticle size distribution; vibrational frequencies of 4-MBA in the Raman and SERS spectra; EF and effective surface area estimation; uniformity of the SERS signal over a 100 × 100 μm^2^ area and substrate contamination; estimation of the parameters for oxygen plasma removal of the 4-MBA molecules attached to the substrate; typical vibrational frequencies of DOX water solution (PDF)
