# Supplementary material for: Stable and Reusable Lace-like Black Silicon Nanostructures Coated with Nanometer-Thick Gold Films for SERS-Based Sensing
Source: ACS Appl Nano Mater. 2023 Mar 9;6(6):4770–81. doi: 10.1021/acsanm.3c00281 (PMC10043874; doi:10.1021/acsanm.3c00281)
Supplement: Supplementary file 1 — an3c00281_si_001.pdf [file an3c00281_si_001.pdf]

Supplementary Material

Stable and Reusable Lace-Like Black Silicon Nanostructures Coated with Nanometer-Thick Gold Films for SERS-Based Sensing

Lena Golubewa[*,1,2], *Aliona Klimovich*[3], *Igor Timoshchenko*[1], *Yaraslau Padrez*[1], *Marina Fetisova*[2], *Hamza Rehman*[2], *Petri Karvinen*[2], *Algirdas Selskis*[4], *Sonata Adomavičiūtė-Grabusovė*[5], *Ieva Matulaitienė*[3], *Arunas Ramanavicius*[6], *Renata Karpicz*[1], *Tatsiana Kulahava*[1], *Yuri Svirko*[2], *Polina Kuzhir*[2]

[1] Department of Molecular Compound Physics, State research institute Center for Physical Sciences and Technology, Sauletekio Av. 3, Vilnius, LT-10257, Lithuania

[2] Department of Physics and Mathematics, Center for Photonics Sciences, University of Eastern Finland, Yliopistokatu 7, Joensuu, FI-80101, Finland

[3] Department of Organic Chemistry, State research institute Center for Physical Sciences and Technology, Sauletekio Av. 3, Vilnius, LT-10257, Lithuania

[4] Department of Characterization of Materials Structure, State research institute Center for Physical Sciences and Technology, Sauletekio Av. 3, Vilnius, LT-10257, Lithuania

[5] Institute of Chemical Physics, Vilnius University, Sauletekio Av. 9, Vilnius, LT-10222, Lithuania

[6] Department of Physical Chemistry, Vilnius University, Naugarduko 24, Vilnius, LT-03225, Lithuania

1. Black silicon absorbance

Absorption spectra of bSi were obtained using the integrating sphere.

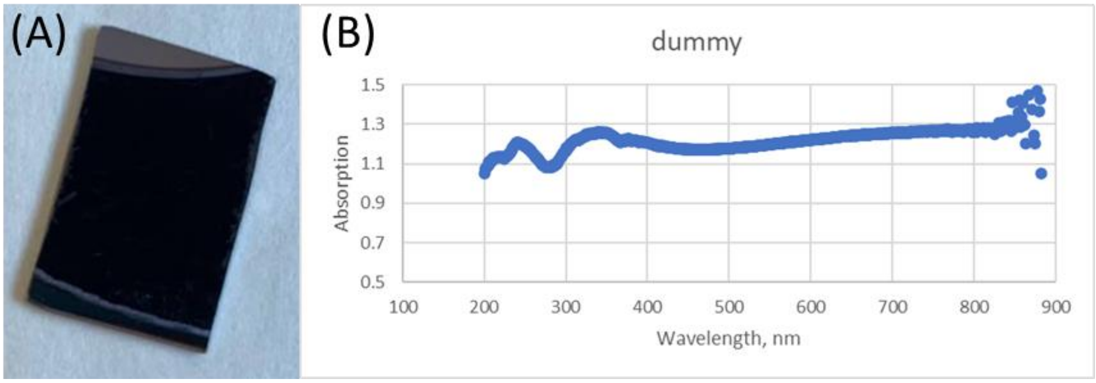

Figure S1. Synthesized bSi photograph (A) and bSi absorption normalized on the absorption values of the unetched silicon measured with integrating sphere (B).

2. Large-scale uniformity and regularity of bSi structures, proved with SEM micrographs of bSi and bSi/Au

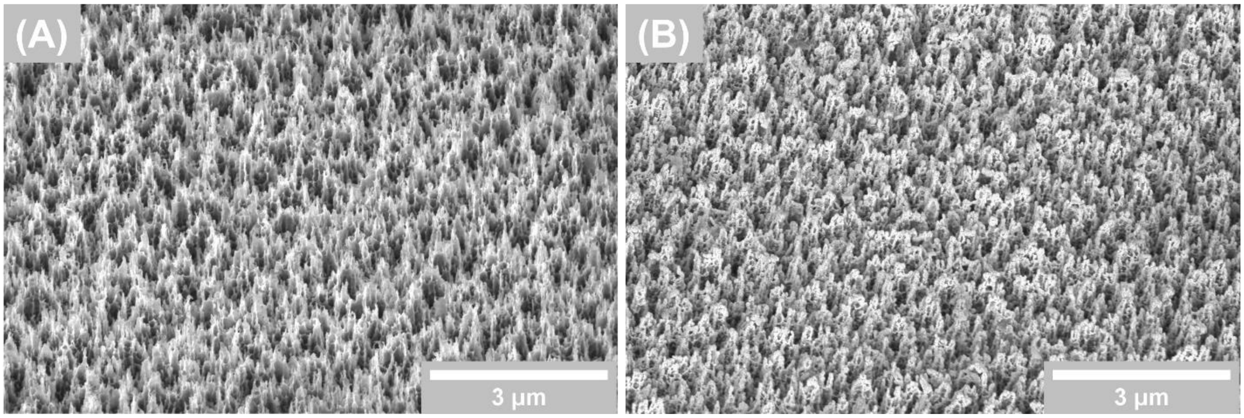

Figure S2. Angle-view SEM images of bSi (A) and bSi sputtered with gold (B). Scanned area is 7 μm × 10 μm).

3. Evaluation of gold nanoparticle size distribution from SEM micrograph analysis

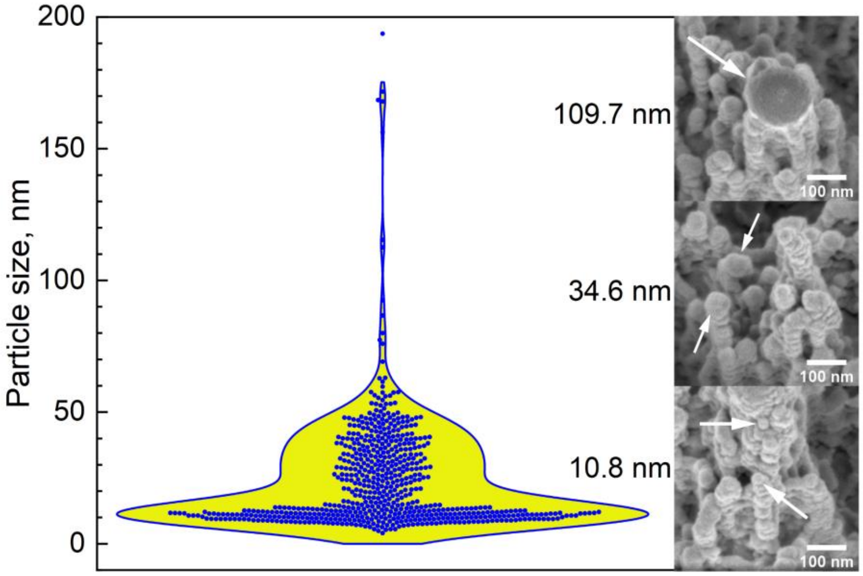

Figure S3. Violin plot showing the size (diameter, nm) distribution of gold nanoparticles on bSi. White arrows indicate typical particles of each group on the bSi surface, the average sizes of which are indicated to the left of the SEM images.

4. Vibrational frequencies of 4-MBA in Raman and SERS spectra

Table S1. 4-MBA band assignments.

4-MBA on bSi/Au	4-MBA (powder)	Assignment	Ref
521 (s)	–	First order optical phonon peak of silicon	
631 (w)	633 (s)	ν_{6b}	S1
		δ(CCC)	S2
668 (w)	685 (w)	β(CO_2)+ ν(CS)	S1
718 (m)	-	γ(CCC) out-of-plane (flat oriented to the surface)	S1, S3
797 (w)	804 (s)	ν_{10a}	S1
		γ(CCC)(CC)	S2
849 (m)	–	β(COO^-)	S1, S3
–	910 (m)	β(SH)	S1, S3
1012 (m)	–	Ring deformation	S1
1076 (vs)	–	ν_{12} – ring breathing	S1
–	1100 (vs)	ν(CC)+δ(CCC)+ν(SC)	S2
–	1119 (w)	ν_{19b}+ ν(CS)	S1
–	1138 (s)	ν_{9a}	S1
		δ(HCC)+ν(CC)	S2
1142 (m)	–	ν($CCOO^-$) + ν(CS)	S1
1178 (m)	1183 (s)	β(CH)	S1
		δ(HCC)+ν(CC)+ν(OC)	S2
–	1294 (s)	ν_3	S1
		δ(HCC)+ν(OC)+δ(HCO)	S2
–	1318 (w)	ν(OC)+δ(HCO)+δ(HCC)	S2
–	1375 (w)	(C-C)	S4
1417 (m)	1405 (m)	ν_s(COO^-)	S3
–	1453 (m)	ν(COO^-)	S1

1481 (m)	1491 (w)	δCH-as	[S5]
1588 (vs)	1595 (vs)	ν_{8a} – ring breathing	[S1]
		ν(CC)	[S2]
–	1623 (m)	ν(C=O)	[S1, S2]
		ν(OC)+δ(HOC)	
–	1652 (w)	ν(OC)	[S2]
1702 (m)	–	ν(C=O)	[S1, S3]
		neutral carboxylic group, COOH are hydrogen bonded	
–	2569 (s)	ν(SH)	[S1, S2, S3]
3074 (w)	3069 (s)	ν(CH) (week for flat oriented molecules)	[S3]

vs – very strong, s – strong, m – medium, w – weak;

ν, stretching; δ, in-plane deformation; γ, out-of-plane deformation.

5. EF and effective surface area estimation

Quantitative evaluation of the effective bSi surface area was performed in Wolfram Mathematica 13. Side-view SEM micrographs were used to estimate the roughness of the bSi.

A typical surface profile of the bSi cross-section is highlighted with a red line in Figure S7, Supplementary Material. Using the assumption that the bSi structure can be represented as a sum of intersecting unidirectional cones, the excess of the effective surface area over its projection area can be replaced by the ratio of the square of the profile length to the square of its projection (or the linear length of the substrate) and it can be estimated according to the formula (S1). At least 5 images of bSi cross-sections with a total length of 20 μm were used.

$$\frac{S_{structure}}{S_{substrates}} \simeq \frac{L^2_{contour}}{L^2_{substrates}} \simeq 22 \qquad \text{(S1)}$$

where $S_{structure}$ – effective surface area of bSi, $S_{substrates}$ – bSi effective surface area projection on the XY plane, $L_{contour}$ – length of the selected profile, $L_{substrates}$ – selected profile projection (linear length of the substrate).

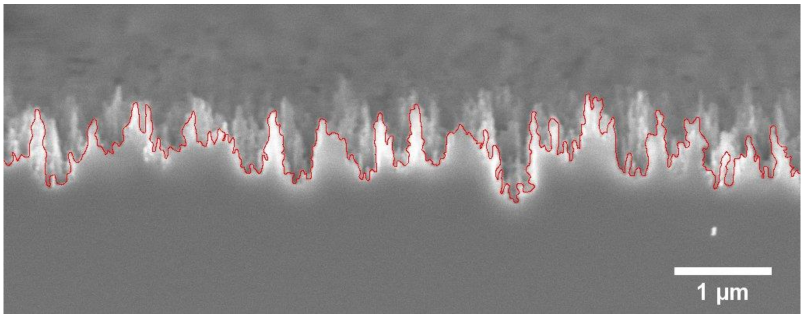

Figure S4. SEM images of the silicon of the cross-section with outlined nanostructures.

To obtain Raman spectra of 4-MBA a droplet of 1.0 mM 4-MBA solution (in ethanol) was placed on Si wafer and dried at room temperature. The procedure was repeated until the characteristic Raman bands became detectable. The total volume of droplets on the Si wafer ($S_{wafer} = 0.792$ cm^2) was 280 μL, droplets were deposited subsequently, waiting for each to dry, the number of molecules in the dried droplets was $N_{In\ Droplets} = 10^{-3}$ mol/L × 2.8 × 10^{-4} L × 6.02 × 10^{23} mol^{-1} = 1.7 × 10^{17}. Thus, estimating that the laser spot diameter was 200 μm, the number of irradiated molecules was N = 6.744 × 10^{13}. Taking into account the effective surface area of the bSi/Au substrate calculated from the SEM micrographs and assuming that a molecule surface density of 4-MBA SAM is equal to 0.5 nmol/cm^2 [S6], the evaluation of the number of the molecules on the bSi/Au substrate gives $N_{bSi/Au} = 9.45 \times 10^{10}$.

Therefore, at the measured intensity ratio of $I_{bSi/Au}/I_{NR} = 1520.1$ for the aromatic ring breathing mode at 1076 cm^{-1}, eq. (1) gives a SERS enhancement factor of EF ≈ 1.1 × 10^6.

6. Uniformity of SERS signal over 100 × 100 µm area and substrate contamination

Table S2. Peak intensity analysis for bands 521 cm^{-1}, 1076 cm^{-1} and 1588 cm^{-1} for 10201 spectra from 2D map

Parameter	521 cm^{-1}	1076 cm^{-1}	1588 cm^{-1}
Mean intensity	3015	11485	6724
Std	194	715	445
Min	2216	8318	5014
25%	2873	11019	6419
50%	3021	11423	6665
75%	3154	11882	6966
Max	4169	17109	10152

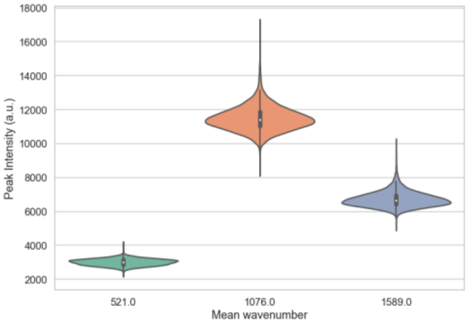

To determine spectra that are considerably different from the mean one the area under the spectra was calculated. Since all spectra have peaks at 521 cm^{-1}, 1076 cm^{-1}, 1588 cm^{-1} the regions 510-540 cm^{-1}, 1050-1100 cm^{-1}, and 1570-1600 cm^{-1} were excluded from computation. The area distribution showed on Fig. S4 A allows detection of the outlying spectra having area greater than Q3 + 1.5 (Q3-Q1), where Q1 and Q3 are 25% and 75% percentile values respectively. These 434 spectra are visualized by green color on Fig. S4B.

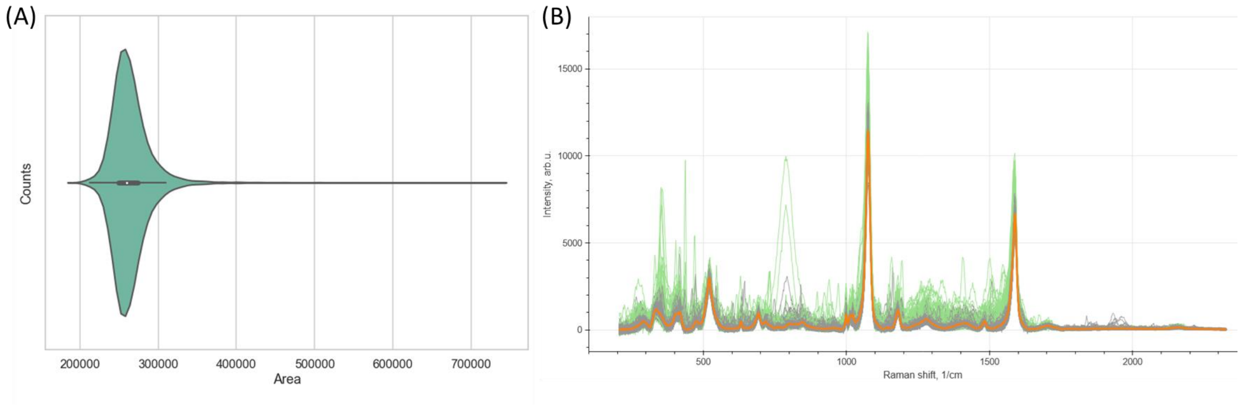

Figure S5. Contamination rate determination: (A) Area under the spectra distribution (521 cm^{-1}, 1076 cm^{-1} and 1588 cm^{-1} bands are excluded), (B) Spectra of contaminating substances (orange line – average spectrum, gray lines – 4-MBA spectra, green lines – spectra of impurities).

7. Estimation of the parameters for oxygen plasma removal of the 4-MBA molecules attached to the substrate

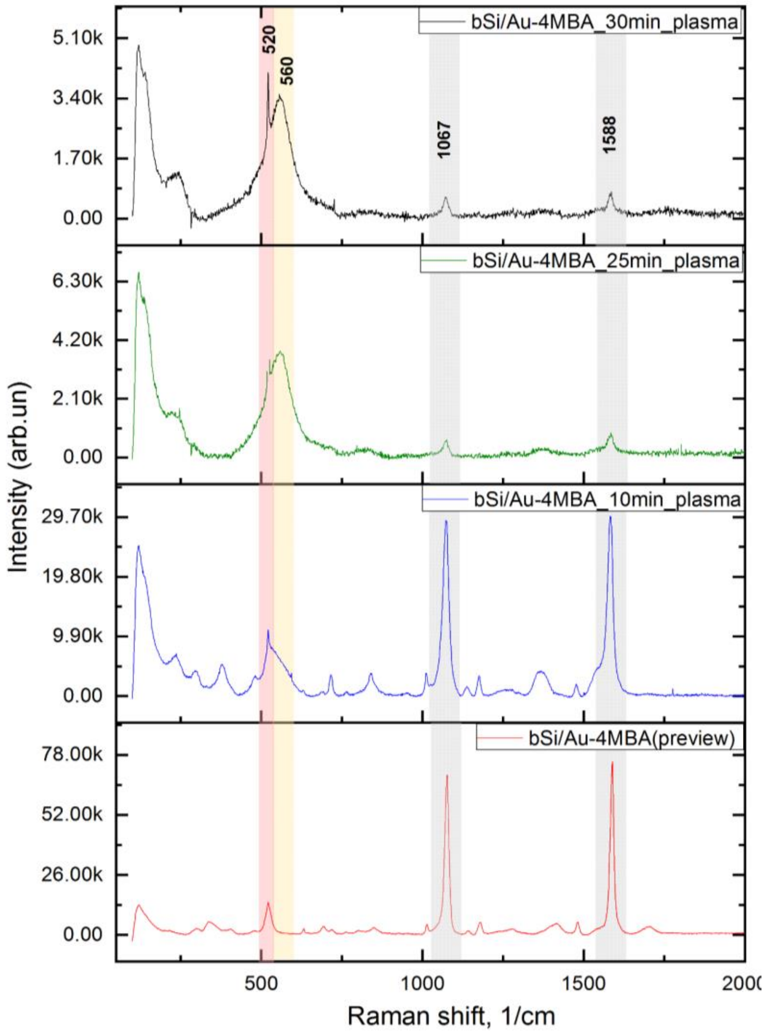

Figure S6. Comparison of the SERS spectra of the 4-MBA monolayer on the bSi/Au substrate during the cleaning process: preview (a), 10 min plasma (b), 25 min plasma (c) and 30 min plasma cleaning(d). The excitation wavelength is 785 nm.

8. Typical vibrational frequencies of DOX water solution

Table S3. Vibrational frequencies (cm^{-1}) of major bands in SERS spectrum of 10^{-5} M DOX solution on bSi/Au substrate [s7]:

**Free DOX, position, cm^{-1}**	**Assignments**
1638 vw*	ν(CO)
1586 sh	Ring stretch + ν(CO)
1576 w	Ring stretch
1564 w	Ring stretch
1456 m	Ring stretch + δ(CC–O)
1434 m	Ring stretch + δ(CC–O)
1413 w	Ring stretch
1343 w	Ring stretch
1296 w	Ring stretch + ν(C–O)
1244 s	Ring stretch symmetry B1 + δ(O–H)
~1224 sh	Ring stretch
1210 s	Ring stretch symmetry A1 + δ(O–H)
~1150 vw	δ(C–H)
1082 w	Skeletal deformation
990 w	Ring breath
917 vw	Skeletal deformation
795 vw	Skeletal deformation
679 vw	Skeletal deformation
504 vw	Skeletal deformation
465 vs	δ(CO)
~450 sh	Skeletal deformation
443 vs	δ(C–O)

*vw – very weak; w – weak; m – medium; s – strong; vs – very strong, sh – shoulder.

REFERENCES

(S1) Capocefalo, A.; Mammucari, D.; Brasili, F.; Fasolato, C.; Bordi, F.; Postorino, P.; Domenici, F. Exploring the Potentiality of a SERS-Active PH Nano-Biosensor. *Front. Chem.* **2019**, *7*, 413. https://doi.org/10.3389/fchem.2019.00413.

(S2) Marques, F. C.; Alves, R. S.; dos Santos, D. P.; Andrade, G. F. S. Surface-Enhanced Raman Spectroscopy of One and a Few Molecules of Acid 4-Mercaptobenzoic in AgNP Enabled by Hot Spots Generated by Hydrogen Bonding. *Phys. Chem. Chem. Phys.* **2022**, *24* (44), 27449–27458. https://doi.org/10.1039/d2cp03375e.

(S3) Michota, A.; Bukowska, J. Surface-Enhanced Raman Scattering (SERS) of 4-Mercaptobenzoic Acid on Silver and Gold Substrates. *J. Raman Spectrosc.* **2003**, *34* (1), 21–25. https://doi.org/10.1002/jrs.928.

(S4) Mhlanga, N.; Ntho, T. A. A Theoretical Study of 4-Mercaptobenzoic Acid Assembled on Ag for Surface-Enhanced Raman Scattering Applications. *Mater. Today Commun.* **2021**, *26*, 101698. https://doi.org/10.1016/j.mtcomm.2020.101698.

(S5) Jiang, L.; You, T.; Yin, P.; Shang, Y.; Zhang, D.; Guo, L.; Yang, S. Surface-Enhanced Raman Scattering Spectra of Adsorbates on Cu2O Nanospheres: Charge-Transfer and Electromagnetic Enhancement. *Nanoscale* **2013**, *5* (7), 2784–2789. https://doi.org/10.1039/c3nr33502j.

(S6) Orendorff, C. J.; Gole, A.; Sau, T. K.; Murphy, C. J. Surface-Enhanced Raman Spectroscopy of Self-Assembled Monolayers: Sandwich Architecture and Nanoparticle Shape Dependence. *Anal. Chem.* **2005**, *77* (10), 3261–3266.

https://doi.org/10.1021/ac048176x.

(S7) Gautier, J.; Munnier, E.; Douziech-Eyrolles, L.; Paillard, A.; Dubois, P.; Chourpa, I. SERS Spectroscopic Approach to Study Doxorubicin Complexes with Fe 2+ Ions and Drug Release from SPION-Based Nanocarriers. *Analyst* **2013**, *138* (24), 7354–7361. https://doi.org/10.1039/c3an00787a.
